# Vitamin D Levels and Length of Hospitalization in Indian Patients With COVID-19: A Single-Center Prospective Study

**DOI:** 10.7759/cureus.26704

**Published:** 2022-07-09

**Authors:** Amrit Dhar, Hyder Mir, Parvaiz A Koul

**Affiliations:** 1 Department of Internal and Pulmonary Medicine, Sher-i-Kashmir Institute of Medical Sciences, Srinagar, IND

**Keywords:** hospitalization, prognosis, covid-19, sars-cov-2, vitamin d

## Abstract

Introduction

The role of vitamin D deficiency in increasing susceptibility or modifying outcomes in severe acute respiratory syndrome coronavirus 2 (SARS-CoV-2) illness is unclear, and data about the association is scant in low- and middle-income countries. We set out to investigate any correlation between baseline vitamin D status and the length of hospital stay in laboratory-confirmed SARS-CoV-2 patients in India.

Methods

Two hundred patients with SARS-CoV-2 infection requiring admission in a North Indian 1200-bedded tertiary care hospital were recruited prospectively from November 2020 to March 2021. Baseline serum 25 hydroxyvitamin D [25(OH)D] levels were measured within 24 hours of admission using chemiluminescent immunoassay. Patients were managed as per hospital management protocol for COVID-19. The primary outcome was the length of hospital stay; secondary outcomes were comparative clinical severity between two groups, rate of requirement of mechanical ventilation and/or non-invasive ventilation (NIV), and mortality. Vitamin D deficiency (VDD) was defined as baseline vitamin D levels of <30 ng/ml.

Results

Of the 200 recruited patients, 57.5% (n = 115) patients were vitamin D deficient, and the overall median length of hospital stay was around 12 days (IQR: 8-15 days). There was no statistically significant difference in the length of hospital stay between patients with normal serum vitamin D (VDS) and those with VDD, median LOS being 12 days (95% CI: 10, 12 days) in VDD cases and 11 days (95% CI: 10,13 days) in VDS cases (p = 0.176). No association between baseline 25(OH)D and any of the secondary outcomes could be established.

Conclusions

In Indian patients, baseline vitamin D levels are not associated with the length of hospital stay, need for mechanical ventilation, or mortality.

## Introduction

Vitamin D plays a crucial role in calcium and phosphorus metabolism and bone remodeling; however, many other functions have emerged, such as its immunomodulatory role in both infectious and autoimmune diseases [1,2]. Vitamin D via regulation of interactions between lymphocytes and antigen-presenting cells enhances the innate immune system and inhibits the adaptive immune system. This action is known to be exerted through effects on both dendritic and T cells [3]. In the setting of vitamin D deficiency (VDD), innate immune functions are impaired, increasing the risk of severe infection. Vitamin D is also known to play a crucial role in maintaining a balance between autophagy and apoptosis to maximize antiviral responses to infection. Thus, it has been suggested as a potential risk factor for many viral infections including HIV, influenza A, rotavirus, hepatitis C, and the severe acute respiratory syndrome coronavirus 2 (SARS-CoV-2) that resulted in the ongoing coronavirus disease-2019 (COVID-19) pandemic [4].

Low serum 25-hydroxyvitamin D [25(OH)D] levels are frequently encountered in individuals with chronic conditions, such as hypertension, diabetes mellitus, malignancies, or cardiovascular diseases, all of which have been reported to be poor prognostic factors for COVID-19 illness [5,6].

Even as several investigators have inquired into the association of vitamin D levels with the SARS-CoV-2 infection and its outcomes, the results have been conflicting. A recent meta-analysis indicated that 25(OH)D can be used as a marker of COVID-19 in-hospital severity and might have a protective role on COVID-19 clinical outcomes [7]; however, in the current scenario, the predictive role of vitamin D status on disease prognosis still remains inconclusive, and no concrete clinical evidence is yet reported [4,8]. Data are scant about the role of the underlying vitamin D status in the length of hospital stay among patients with COVID-19. One recent study demonstrated VDD to be a factor for increased risk of hospitalization from COVID-19 illness [9], whereas another Brazilian study showed that among hospitalized patients with COVID-19 illness, a trend for longer hospital stay was seen in patients with VDD [10].

The current study was designed to study the relationship between the length of hospitalization and outcomes of COVID-19 illness and the baseline vitamin D levels at admission, keeping in mind an association of viral illnesses like influenza with VDD [11].

## Materials and methods

This was a single-center prospective cohort study performed between November 2020 and March 2021 in a 1200-bedded Tertiary, University Teaching Hospital in North India.

A total of 200 adult (age > 18 years) patients (median age: 64.5 years; IQR: 54-71.25 years), with 60% (n = 120) males and 40% (n = 80) females, with COVID-19 illness, confirmed via reverse transcriptase-polymerase chain reaction (RT-PCR) for SARS-CoV-2 by nasopharyngeal swabs, were enrolled in the study within 24 hours of their admission. Patients who were unwilling to participate in this study, pregnant and lactating women, and those with recent supplementation with vitamin D in the past two months or less were excluded.

Patients who were enrolled were immediately followed up until the day of discharge or death. Data regarding demographics, clinical history, co-existing chronic diseases, and prior vitamin D supplementation were collected and analyzed, and other baseline laboratory investigations (hemogram, kidney function, liver function, serum calcium, and serum phosphorous) were collected. Inflammatory markers (IL-6, C-reactive protein [CRP], ferritin, and D-dimer) were also analyzed. For vitamin D status, the blood sample was collected in a clot activator vacutainer and sent for serum 25(OH)D analysis. Serum 25(OH)D was quantified by chemiluminescent immunoassay (Automated Beckman Coulter Access 2 Immunoassay System, Beckman Coulter Inc., USA). All the samples were analyzed at the same time in the same laboratory (Immunology laboratory of Sher-i-Kashmir Institute of Medical Sciences, India).

All patients received a standard treatment consisting of oxygen supplementation (as required), antivirals, and steroids as per the institutional protocol. Antibiotics were reserved for cases with features suggestive of bacterial co-infection or secondary infection during hospitalization.

The study was approved by the Institutional Ethics committee of Sher-i-Kashmir Institute of Medical Sciences (IEC SKIMS) with the approval number # RP 06/2021), and all procedures were in accordance with the declaration of Helsinki. The participants were enrolled after written informed consent.

Outcome measures

The primary outcome was the length of hospital stay, defined as the total number of days that a patient remained hospitalized from date of admission to date of discharge, the discharge criteria being: no fever in the last 72 hours or more; maintaining oxygen saturation > 92% without respiratory distress; and minimal oxygen demand of 1-2 liter/minute, to be delivered via domiciliary oxygen, if available. These criteria were in accordance with the local institutional protocols. The secondary outcomes were comparative clinical severity of COVID-19, overall mortality, overall requirement for non-invasive or invasive mechanical ventilation between the two categories, and correlation of various inflammatory markers with baseline vitamin D levels. Patients were categorized as those with vitamin D sufficiency (VDS) (≥30 mg/mL) and VDD (<30 ng/mL) [12].

Statistical analysis

For descriptive statistics, the continuous variables were summarized as mean + SD (standard deviation) or median and IQRs as appropriate. The categorical variables are depicted as frequencies and percentages. All data were assessed for normality. Student’s t-test, Fisher's exact test, and Mann-Whitney U test were used wherever applicable. Kaplan-Meir analysis for VDD and VDS were compared using the log-rank test. Pearson’s correlation was done for continuous variables. Multivariable Cox proportional hazard analysis was used adjusting for age, sex, body mass index (BMI), hypertension, diabetes mellitus, and chronic obstructive pulmonary disease (COPD).

Statistical analysis was done using the SPSS (Statistical Package for the Social Sciences) software, version 28.0 (IBM Corp., Armonk, NY). The significance level was set at p < 0.05.

## Results

Overall, 512 COVID-19 patients were admitted during our study period of which 200 consenting cases were included after applying eligibility criteria. Overall, the mean age of the study group was 61.21 ± 12.32 (range: 18-90) years. The mean levels of serum vitamin D [25(OH)D] levels were 32.4 ± 26.4 (range: 1.41-165.69) ng/mL. Table 1 presents the baseline demographic and clinical characteristics in patients with COVID-19 according to 25(OH)D categories (VDD or VDS). Both the groups were quite similar to each other in view of demographic characteristics. Hypertension was significantly more prevalent in the VDD group, whereas diabetes mellitus was also seen more frequently in this group. Overall concentrations of the inflammatory markers also did not differ in the two groups.

**Table 1:** Characteristics of enrolled COVID-19 patients according to serum 25(OH)D categories C-reactive protein was not included as quantitative data. The qualitative assessment was not significant. IQR: Interquartile range; SD: Standard deviation; BMI: Body mass index; COPD: Chronic obstructive pulmonary disease; IL-6; Interleukin-6; ICU: Intensive care unit. For continuous variables, the student's t-test and Mann-Whitney U test were used; for categorical variables, the Fischer's exact test and chi-square test were used.

Variables	25(OH)D < 30 ng/mL; n = 115	25(OH)D ≥ 30ng/mL; n = 85	p-values
Baseline characteristics			
Age (years), median (IQR)	63.0 (50.0-73.0)	65.0 (55.0-70.0)	0.58
Sex, n (% within stratum)			
Male	74 (64.3)	46 (54.1)	0.148
Female	41 (35.7)	39 (45.9)	
BMI (kg/m^2^), mean ± SD	28.57 ± 3.87	28.32 ± 4.18	0.65
Hypertension, n (%)	60 (52.1)	62 (72.9)	0.003
Diabetes mellitus, n (%)	40 (34.7)	35 (41.1)	0.188
COPD, n (%)	18 (15.6)	16 (18.8)	0.57
Cardiovascular disease, n (%)	65 (56.5)	39 (45.9)	0.15
Malignancy, n (%)	7 (6.08)	5 (5.88)	1.00
Laboratory data			
Total leucocyte count (mm^3^), median (IQR)	7600 (5200-10 600)	7900 (5750-11 300)	0.406
Platelet count (lakhs/mm^3^), median (IQR)	139 (110-190)	149 (103-203)	0.449
Serum ferritin (ng/mL), median (IQR)	381 (277-586)	341 (303-610)	0.815
D-dimer (ng/mL), median (IQR)	397 (239-976)	361 (182-605)	0.089
IL-6 (pg/mL), median (IQR)	17.69 (6.3-47.90)	13.42 (5-47.70)	0.27
Corrected calcium (mg/dL), mean ± SD (Range, mg/dL)	8.94 ± 0.66 (7.1-12.7)	9.11 ± 0.59 (7.9-12)	0.07
Phosphorous (mg/dL), mean ± SD (Range, mg/dL)	3.06 ± 2.8 (1.33-5.91)	3.04 ± 0.96 (1.11-7.29)	0.88
25(OH)D (ng/mL), mean ± SD (Range, mg/dL)	15.65 ± 8.40 (1.41-29.82)	55.19 ± 25.45 (30.21-165.29)	<0.0001
Severity of illness			
Mild, n (%)	12 (10.5)	10 (11)	0.916
Moderate, n (%)	11 (9.5)	7 (8)	
Severe/Critical, n (%)	92 (80)	68 (85)	
Outcomes			
ICU admission, n (%)	33 (29)	27 (31)	0.643
Mechanical ventilation/Non-invasive ventilation, n (%)	31 (26)	24 (28)	0.87
Death, n (%)	24 (20)	23 (27)	0.31
Length of hospital stay (days), median (95% CI)	12 [10,12]	11 [10,13]	0.5541

Primary outcome

**Figure 1:**
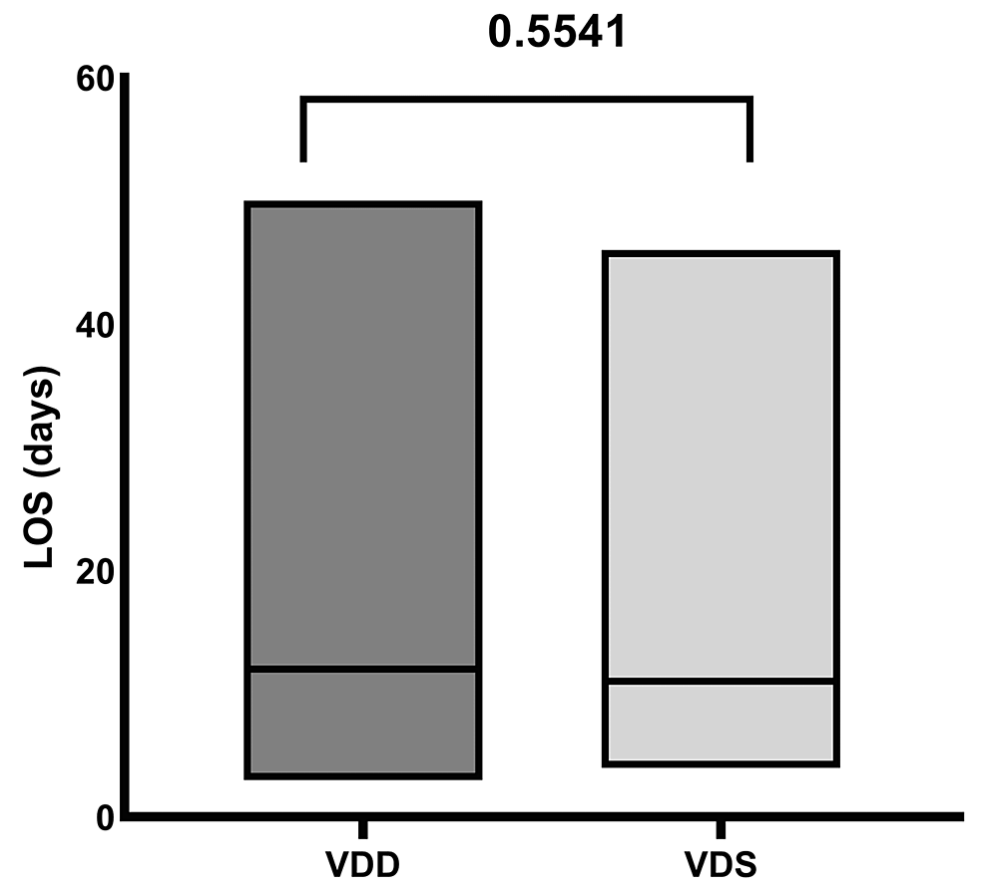
Bar plot representing LOS (days) of patients with VDD and VDS in patients with COVID-19 (p = 0.5541) VDD: Vitamin D deficiency; VDS: Vitamin D sufficiency; LOS: Length of stay.

The median length of hospital stay overall was 12 days (IQR: 8-15 days). It was found to be similar with a median of 12 days (95% CI: 10,12 days) in VDD and 11 days (95% CI: 10,13 days) in VDS (p = 0.5541) as shown in Figure 1. There was no obvious trend for longer length of hospital stay in patients with severe COVID-19 illness.

**Figure 2:**
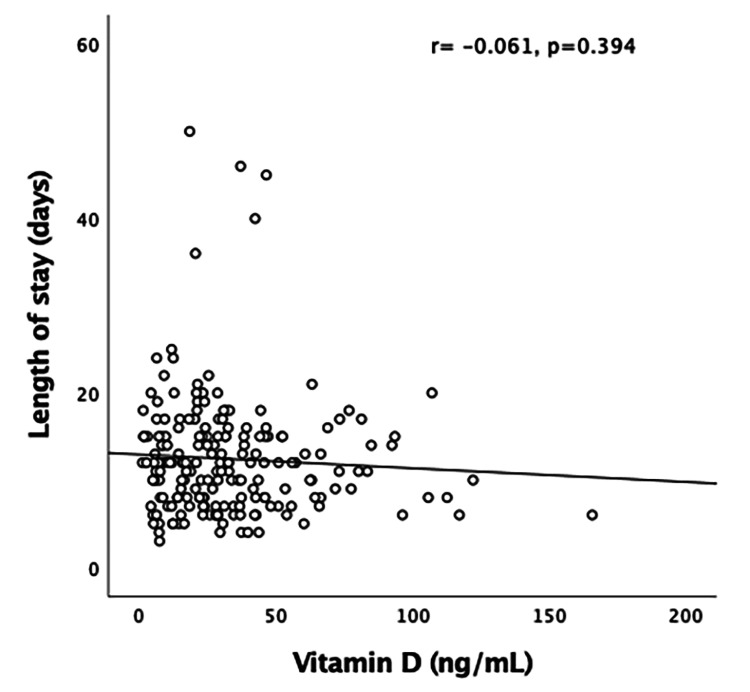
Pearson’s correlation of vitamin D level with LOS (days) LOS: Length of stay.

Figure 2 represents a minimal negative correlation, which was non-significant (r = -0.061, p = 0.394).

**Figure 3:**
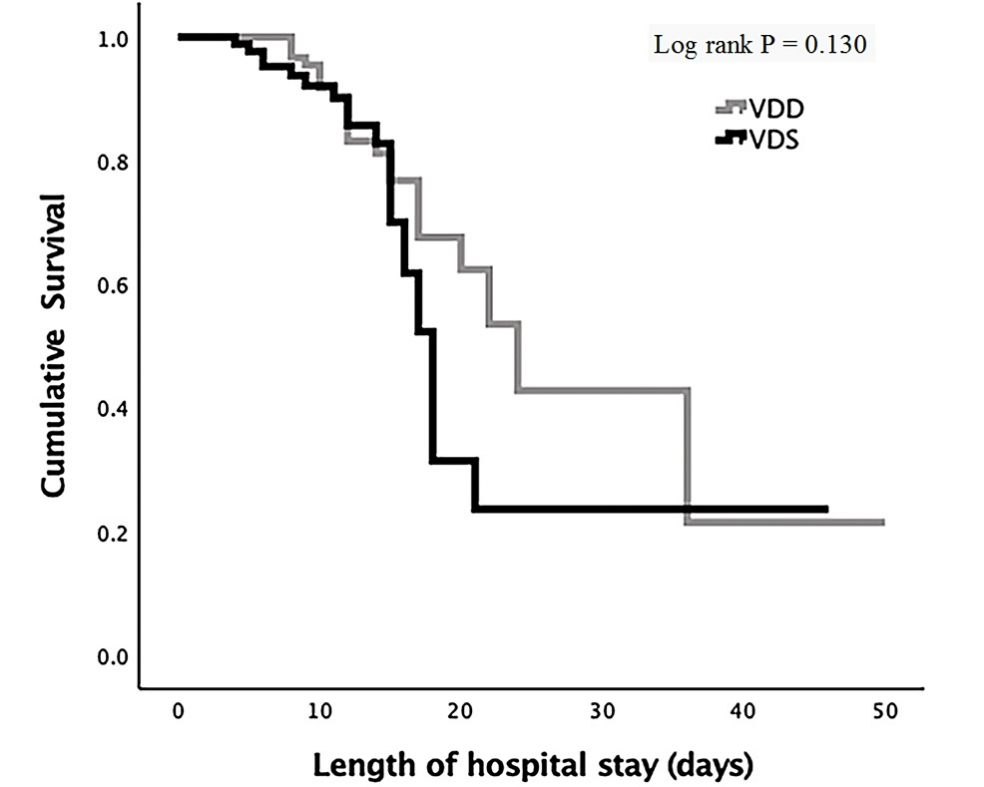
Kaplan-Meier curves for hospital length of stay according to 25-hydroxyvitamin D categories VDD vs VDS. Vertical lines represent single censored events (deaths). VDD: Vitamin D deficiency; VDS: Vitamin D sufficiency.

The Kaplan-Meier survival analysis (log-rank p = 0.130) between the two categories did not reveal any survival benefit for patients with sufficient vitamin D (Figure 3).

Table 2 depicts a multivariate Cox regression model for patients with VDS and VDD, respectively, for the length of hospital stay. Patients with no hypertension in the VDS subgroup had a higher chance of hospital discharge than hypertensives.

**Table 2:** Results of multivariate Cox regression model for patients with VDS and VDD for primary and secondary outcomes VDD: Vitamin D deficiency; VDS: Vitamin D sufficiency; BMI: Body mass index; COPD: Chronic obstructive pulmonary disease; HR: Hazard ratio. * indicates a significant p-value (<0.05).

Covariate	25(OH)D < 30 ng/mL (VDD)		25(OH)D ≥ 30 ng/mL (VDS)	
	HR (95% CI)	p-value	HR (95% CI)	p-value
Hospital discharge/Length of hospital stay				
Age	0.98 (0.97, 0.99)	0.037 (*)	0.98 (0.97, 1.0)	0.23
Male sex	0.68 (0.43, 1.09)	0.11	0.57 (0.32, 1.01)	0.06
BMI	1.0 (0.95, 1.06)	0.84	0.95 (0.89, 1.02)	0.218
Hypertension	0.69 (0.43, 1.12)	0.13	0.39 (0.20, 0.76)	0.006 (*)
Diabetes mellitus	0.91 (0.57, 1.45)	0.69	1.42 (0.75, 2.68)	0.27
COPD	1.0 (0.48, 2.08)	0.98	0.56 (0.16, 1.92)	0.35
Mechanical ventilation and/or non-invasive ventilation				
Age	1.02 (0.98, 1.05)	0.19	1.02 (0.98, 1.06)	0.25
Male sex	0.99 (0.41, 2.34)	0.98	1.15 (0.48, 2.76)	0.75
BMI	0.98 (0.87, 1.10)	0.76	1.04 (0.93, 1.16)	0.41
Hypertension	0.41 (0.17, 0.99)	0.049 (*)	1.61 (0.43, 6.02)	0.47
Diabetes mellitus	0.95 (0.38, 2.35)	0.92	0.80 (0.33, 1.96)	0.63
COPD	0.92 (0.30, 2.81)	0.89	1.23 (0.35, 4.31)	0.73
Mortality				
Age	1.01 (0.98-1.05)	0.28	1.02 (0.98, 1.07)	0.18
Male sex	0.88 (0.33-2.33)	0.79	1.04 (0.42, 2.60)	0.92
BMI	0.93 (0.81-1.07)	0.33	1.03 (0.91, 1.16)	0.60
Hypertension	0.30 (0.11-0.85)	0.02 (*)	1.57 (0.40, 6.08)	0.50
Diabetes mellitus	0.77 (0.27-2.18)	0.63	0.73 (0.29, 1.88)	0.52
COPD	0.46 (0.09-2.19)	0.33	1.30 (0.36, 4.68)	0.68

Secondary outcomes

On categorization of the patients on the basis of vitamin D deficiency, out of 200 patients, 115 patients were found to be deficient in vitamin D among which 92 had severe/critical COVID-19 illness, 12 had mild, and 11 had moderate [13]. Among VDS patients, 68 had severe/critical disease, 10 had mild, and seven had moderate disease (p = 0.916).

**Figure 4:**
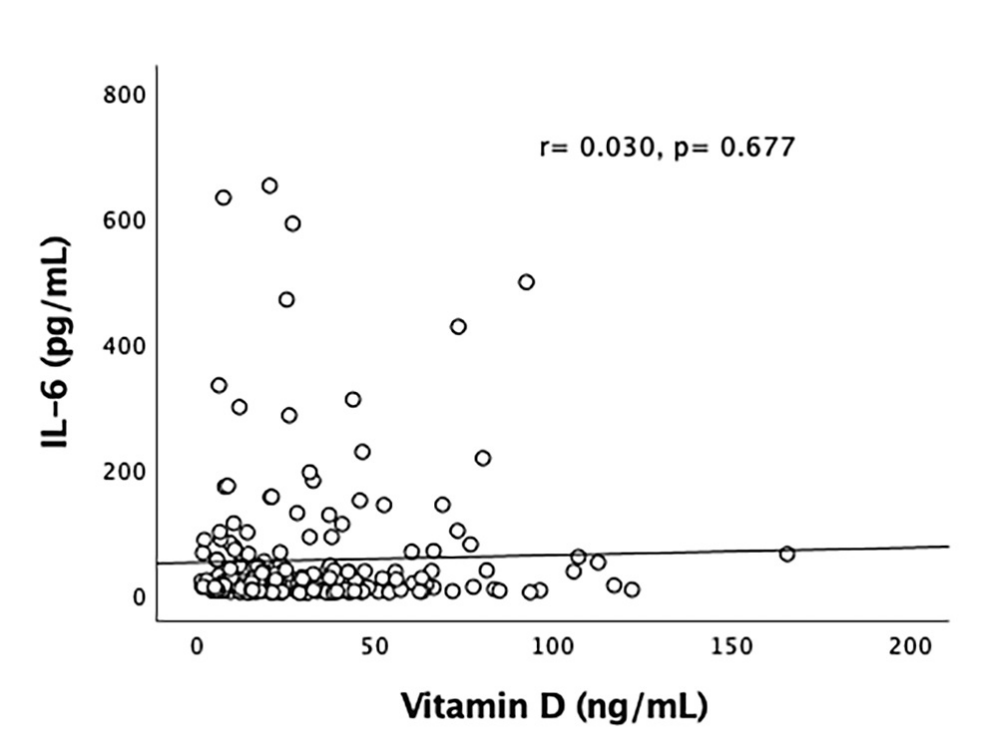
Pearson’s correlation of vitamin D (ng/mL) with IL-6 (pg/mL)

**Figure 5:**
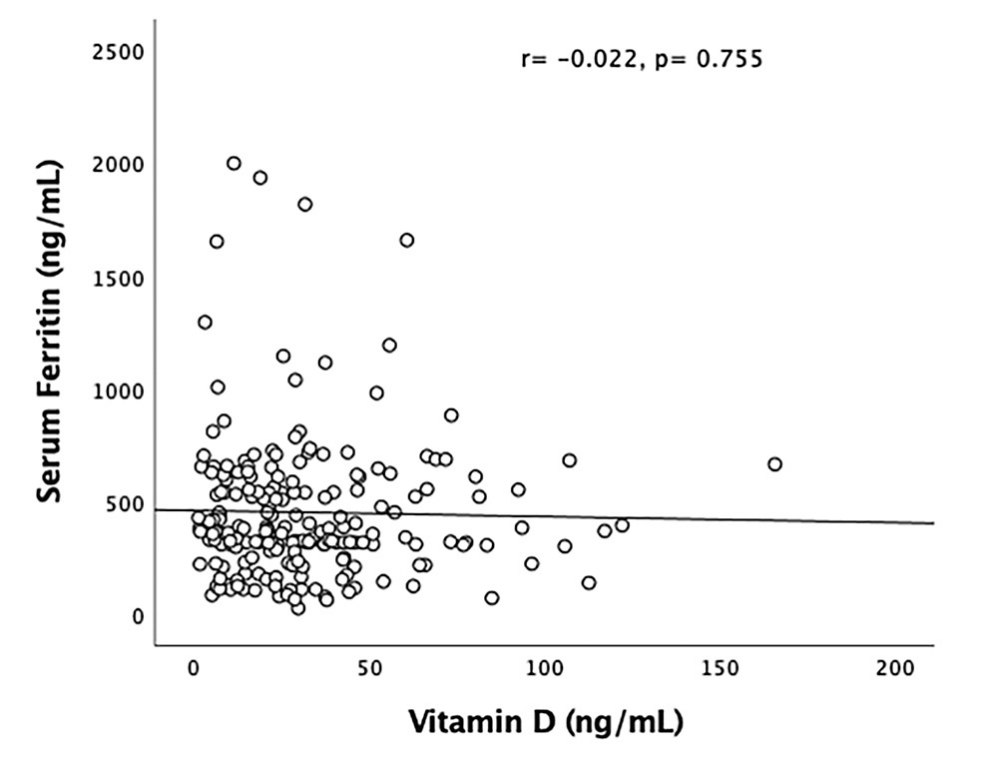
Pearson’s correlation of vitamin D level (ng/mL) with serum ferritin (ng/mL)

**Figure 6:**
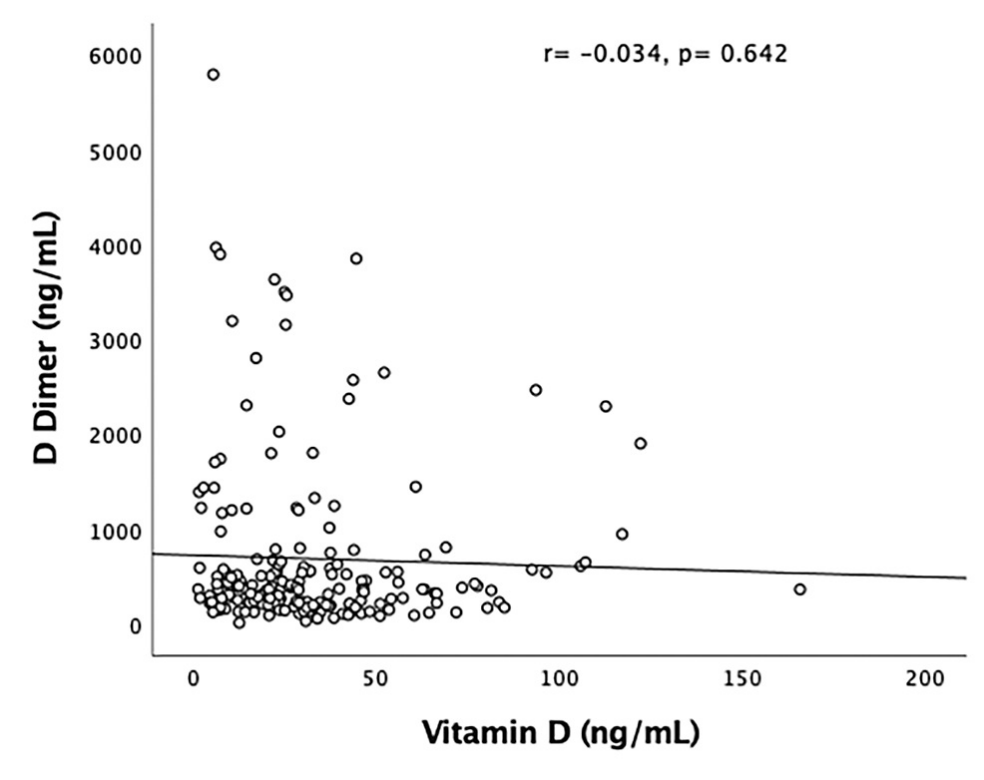
Pearson’s correlation of vitamin D level (ng/mL) with D-dimer (ng/mL)

The number of patients who required mechanical ventilation and/or non-invasive ventilation (NIV) was not significantly different between the two groups. Out of all VDD cases, 26% required eventual mechanical ventilation and/or NIV, whereas 28% of patients with VDS required the same. Mortality was comparable among the two categories, with no statistical significance between them. Overall, 55% of patients with mortality belonged to the VDD category. Figures 4-6 represent the correlation of vitamin D levels with inflammatory markers (IL-6, serum ferritin, and D-dimer). No significant correlation of vitamin D levels with various inflammatory markers for COVID-19 was found. Table 2 depicts the multivariable Cox proportional hazard model.

## Discussion

Ours is the first prospective, cohort study evaluating the association of serum 25(OH)D levels with the length of hospital stay and other clinical outcomes in patients with COVID-19 illness from the Indian subcontinent, a region of endemic VDD [14]. The present study demonstrated that VDD at baseline (defined as 25(OH)D <30 ng/mL) in subjects with COVID-19 illness did not result in adverse outcomes of the disease in relation to the length of hospital stay, the requirement for mechanical ventilation, or mortality rate. Further, no correlation was found between VDD and clinical severity of the illness or elevation of the inflammatory markers. Only one multicenter, prospective cohort study from Sao Paulo, Brazil, has previously addressed the issue of the effect of baseline vitamin D level on the length of hospital stay in admitted patients with SARS-CoV-2 infection where the results were similar to our study and the authors concluded that even though severe 25(OH)D deficiency (defined as 25(OH)D < 10 ng/mL) was associated with a trend for longer hospital stays, the trend was not statistically significant in multivariable Cox regression models and when different cut-offs (25(OH)D >10 to <20 ng/mL, 20 to <30 ng/mL and > 30 ng/mL) were defined [10].

Vitamin D and its protective role in various viral illnesses such as influenza, HIV, and hepatitis C virus is backed by compelling evidence and is an area of growing research interest [15]. Various mechanisms such as induction of apoptosis and autophagy by producing antimicrobial peptides and even genetic and epigenetic factors have been reported as its antiviral effects [4,16]. SARS-CoV-2 virus has been known to induce high angiotensin II production resulting in widespread systemic inflammation. The resultant cytokine storm is postulated to be mediated by the release of several cytokines that, among others, include the tumor necrosis factor-alpha (TNF-α), interleukin-1 beta (IL-1β), and interleukin-6 (IL-6) [17]. In this sense, experimental evidence indicates that vitamin D via its action of attenuating p38 MAP kinase in human macrophages/monocytes inhibits IL-6 and TNF-α. Moreover, vitamin D has postulated a role via inhibition of pro-inflammatory cytokines via induction of T regulatory cells. All of this underlines the possibility that VDS could elicit antiviral effects, which eventually help in the early recovery of COVID-19 patients [18].

The current literature on vitamin D in COVID-19 is neither compelling nor conclusive to any extent. Many retrospective studies have observed lower 25(OH)D levels were associated with poor prognosis in COVID-19 [19,20]. Recently, Ilie et al. found a crude association between vitamin D levels and the number of COVID-19 cases across 20 European countries, the majority of cases including elderly patients with multiple comorbidities [21]. One study from the Indian subcontinent found a strong association between vitamin D status and clinical severity [22]. In contrast, some studies found no association between 25(OH)D and clinical outcomes in patients with COVID-19 (including the need for ICU admission, need for mechanical ventilation, or mortality) [8,23]. This was similar to the findings in our study, where VDS did not translate into reduced hospital stay or mortality. The reason for such contrasting findings could be partially explained by the observational design of studies hampering the establishment of causation. Second, on analysis of different studies, it was found that different cut-offs were used for defining VDD. Also, vitamin D is expected to be lower in elderly and hospitalized patients with multiple comorbidities, which constitutes the majority of patients hospitalized with COVID-19 [24,25].

It is worth mentioning that serum 25(OH)D is considered a negative acute-phase reactant, and its levels are reported to be low during acute inflammatory diseases [26]. Our study, like many others, was carried out in hospitalized patients; thus, in that sense, 25(OH)D levels are expected to be low. Therefore, establishing an association becomes an uphill task, and caution is needed in interpreting vitamin D levels in such a situation. In one study in which pre-hospital 25(OH)D levels were studied, the authors did not find a relation between pre-hospitalization vitamin D levels and clinical outcomes [27]. On the contrary, another study concluded that pre-infection deficiency of vitamin D was associated with increased disease severity and mortality [28]. Finally, a randomized double-blind clinical trial found that among the hospitalized patients with COVID-19, a single high dose of vitamin D3, compared with a placebo, did not significantly reduce the hospital length of stay or any other relevant outcomes [29]. However, as doubtful as its role in treatment for COVID-19 may appear, it is crucial to note that hospitalized patients include elderly, frail individuals with multiple comorbidities and may require immobilization for prolonged periods. In addition, these patients are usually exposed to systemic corticosteroids as a part of treatment for severe COVID-19. All these factors could compound the risk for fractures in these patients. Therefore, low-dose maintenance vitamin D as a preventive strategy has been recommended for such individuals [30]. Therefore, it is pertinent that further randomized clinical trials be undertaken to clarify the potential role of vitamin D on various aspects of COVID-19 illness.

Our study was limited by an observational design, relatively small numbers, and it being a single-center study from an endemically VDD region [31]. However, it is the first study to address the issue of the length of hospitalization in Indian settings and calls for further studies on larger samples to arrive at more statistically robust conclusions.

## Conclusions

In conclusion, our study did not reveal any association of vitamin D sufficiency with reduced length of hospital stay, and no relation with reduced mortality or need for mechanical ventilation could be established. Whether vitamin D plays a role as a treatment modality or in improving prognosis in patients with COVID-19 needs further elucidation in large randomized control trials. However, considering the underlying comorbidities and the prevalence of VDD in a region, giving vitamin D supplements as maintenance for the prevention of fragility fractures can be considered according to the discretion of the treating physician against the backdrop of historical local vitamin D data and institutional protocols.
